# Association between inflammatory cytokines and anti-SARS-CoV-2 antibodies in hospitalized patients with COVID-19

**DOI:** 10.1186/s12979-022-00271-2

**Published:** 2022-03-05

**Authors:** Xixi Jing, Min Xu, Deye Song, Tingting Yue, Yali Wang, Pan Zhang, Yanjun Zhong, Min Zhang, Tommy Tsan-Yuk Lam, Nuno Rodrigues Faria, Erik De Clercq, Guangdi Li

**Affiliations:** 1 Hunan Provincial Key Laboratory of Clinical Epidemiology, Xiangya School of Public Health, Central South University, Changsha, 410078 China; 2 Department of Critical Care Medicine, The Second Xiangya Hospital, Central South University, Changsha, 410011 China; 3 Department of Orthopedics, The Second Xiangya Hospital, Central South University, Changsha, 410011 Hunan China; 4 Institute of Hepatology and Department of Infectious Diseases, The Second Xiangya Hospital, Central South University, Changsha, 410011 Hunan China; 5 State Key Laboratory of Emerging Infectious Diseases, School of Public Health, The University of Hong Kong, Hong Kong, SAR China; 6 Department of Infectious Disease Epidemiology, School of Public Health, Imperial College London, London, UK; 7 Department of Zoology, University of Oxford, Oxford, UK; 8 Department of Microbiology, Immunology and Transplantation, Rega Institute for Medical Research, KU Leuven, 3000 Leuven, Belgium

**Keywords:** SARS-CoV-2, COVID-19, IgG, IgM, Cytokine dynamics

## Abstract

**Background:**

COVID-19 patients may experience “cytokine storm” when human immune system produces excessive cytokines/chemokines. However, it remains unclear whether early responses of inflammatory cytokines would lead to high or low titers of anti-SARS-CoV-2 antibodies.

**Methods:**

This retrospective study enrolled a cohort of 272 hospitalized patients with laboratory-confirmed SARS-CoV-2. Laboratory assessments of serum cytokines (IL-2R, IL-6, IL-8, IL-10, TNF-α), anti-SARS-CoV-2 IgG/IgM antibodies, and peripheral blood biomarkers were conducted during hospitalization.

**Results:**

At hospital admission, 36.4% patients were severely ill, 51.5% patients were ≥ 65 years, and 60.3% patients had comorbidities. Higher levels of IL-2R and IL-6 were observed in older patients (≥65 years). Significant differences of IL-2R (week 2 to week ≥5 from symptom onset), IL-6 (week 1 to week ≥5), IL-8 (week 2 to week ≥5), and IL-10 (week 1 to week 3) were observed between moderately-ill and severely ill patients. Anti-SARS-CoV-2 IgG titers were significantly higher in severely ill patients than in moderately ill patients, but such difference was not observed for IgM. High titers of early-stage IL-6, IL-8, and TNF-α (≤2 weeks after symptom onset) were positively correlated with high titers of late-stage IgG (≥5 weeks after symptom onset). Deaths were mostly observed in severely ill older patients (45.9%). Survival analyses revealed risk factors of patient age, baseline COVID-19 severity, and baseline IL-6 that affected survival time, especially in severely ill older patients.

**Conclusion:**

Early responses of elevated cytokines such as IL-6 reflect the active immune responses, leading to high titers of IgG antibodies against COVID-19.

**Supplementary Information:**

The online version contains supplementary material available at 10.1186/s12979-022-00271-2.

## Introduction

Severe acute respiratory syndrome coronavirus 2 (SARS-CoV-2) is the aetiological agent of coronavirus disease 2019 (COVID-19), causing severe pneumonia, multiorgan dysfunction, or even death [1]. During SARS-CoV-2 infection, a large family of cytokines and chemokines are produced by host cells to initiate inflammatory responses and to mediate innate immune responses [2, 3]. Although most COVID-19 patients are asymptomatic or have mild signs [4, 5], those with severe disease may experience a hyperinflammatory state called a “cytokine storm” in which the immune system produces excessive inflammatory cytokines/chemokines that may cause acute respiratory distress, pulmonary edema, and multiorgan failure [6–9].

Previous studies suggest that serum levels of cytokines and chemokines are likely associated with the disease severity and clinical outcomes of COVID-19 [10–19]. Many COVID-19 patients have been diagnosed with abnormal production of serum cytokines and chemokines such as interleukin-6 (IL-6) [10, 19], IL-1α [11], IL-1β [12], IL-2 receptor (IL-2R) [13], IL-3 [14], IL-7 [20], IL-8 [15], IL-10 [21], IL-13 [22], IL-18 [12], IL-37 [18], tumor necrosis factor-α (TNF-α) [19] and transforming growth factor-β [23], especially in severe and critical cases.

Among the long list of cytokines/chemokines, IL-6 has been recognized as a key COVID-19-associated cytokine. First, IL-6 may serve as an early biomarker to monitor inflammatory and immune responses in COVID-19 patients. Early diagnosis of serum IL-6 can be achieved within the first 10 days after symptom onset; other cytokines are often detected at the later stage [2]. The early responses of elevated IL-6 expression might drive activation of T cell immunity against COVID-19 [24]. Second, baseline IL-6 is a promising predictor of disease severity and mortality during hospitalization of COVID-19 patients [19]. Elevated production of IL-6 in severe cases is likely associated with reduced levels of granzyme A-expressing natural killer cells, indicating impaired immune cell cytotoxicity [25]. Even at 8 months after viral infection, inflammatory biomarkers such as IL-6, interferon-β, and interferon-λ2/3 remain persistently high in COVID-19 survivors [26]. Third, IL-6 has been identified as a COVID-19-associated cytokine by many independent studies [10, 11, 24, 27], while other biomarkers have not been consistently reported in the literature. Overall, elevated expression of serum IL-6 is associated with adverse clinical outcomes and mortality in COVID-19 patients [19], and the IL-6 receptor antagonists such as tocilizumab may reduce 28-day all-cause mortality in hospitalized patients with COVID-19 [28]. Moreover, higher expressions of epithelial cell-derived IL-6 and myeloid-derived IL-1β in lung tissues are distinguishing features of COVID-19 compared with healthy controls, influenza pneumonia, bacterial pneumonia, and acute respiratory distress syndrome [29]. Despite this knowledge, it remains unclear to date whether serum levels of cytokines such as IL-6 are associated with anti-SARS-CoV-2 antibody responses.

During the innate immune response to SARS-CoV-2 infection, inflammatory cytokines are produced at the early stage [30]. Later, emergence of adaptive immune responses leads to production of anti-SARS-CoV-2 antibodies, such as IgM and IgG, which ideally deliver long-term protection [31]. Although the importance of IgM and IgG antibodies is known, it remains unclear whether the late-stage responses of anti-SARS-CoV-2 IgG/IgM antibodies could be predicted by the early responses of inflammatory cytokines. To investigate this hypothesis, we conducted this retrospective study. To avoid the impact of different vaccines, cytokine inhibitors and circulating SARS-CoV-2 variants, we evaluated a cohort of laboratory-confirmed COVID-19 patients who received neither vaccines nor cytokine inhibitors.

## Methods

### Study design and participants

We performed a retrospective study to collect cytokine and antibody records of hospitalized COVID-19 patients during the early outbreak of the pandemic (January to March 2020) when approved vaccines and cytokine-targeted drugs were not available or administered. We analyzed a cohort of 272 COVID-19 patients who were hospitalized from 2020/02/01 to 2020/03/31 in the Sino-French New-City Tongji Hospital in Wuhan, China. At hospital admission, all patients were confirmed to have SARS-CoV-2 infection based on real-time RT–PCR tests of nasopharyngeal swab specimens. During their hospitalization, all patients received neither vaccines nor cytokine-targeted drugs, according to the COVID-19 guidelines before 2020 April. Archived records during the inpatient hospital stay were analyzed in this retrospective study; thereby, written informed consent was waived. This study was conducted under the Helsinki Declaration and was approved by the Ethics Committees of The Second Xiangya Hospital (approval ID: LYF2020060).

Based on the New Coronavirus Diagnosis and Treatment Guidelines in China, a severely ill case was identified if any of the following five conditions were fulfilled at baseline or during hospitalization: (i) the breathing rate of ≥30 breaths per minute; (ii) oxygen saturation of ≤93% at rest; (iii) PaO_2_/FiO_2_ (artery partial pressure of oxygen / inspired oxygen fraction) ≤300 mmHg; (iv) chest imaging tests that exhibit > 50% progression of lung lesions within 48 h; and (v) shock or organ failure that requires intensive care unit, or respiratory failure that requires mechanical ventilation. Moderately ill cases were defined based on mild/moderate symptoms of fever, respiratory difficulties, and/or radiological findings of pneumonia.

### Data collection

Electronic medical records of COVID-19 patients were retrieved to collect datasets of epidemiological and demographic information, laboratory biomarkers, onset symptoms, disease severity and clinical comorbidities at hospital admission. All medical records were retrieved based on a standard collection form from the Sino-French New-City Tongji Hospital. Two authors (XJ, TY) cross-checked the medical records and communicated with doctors for data accuracy.

### Laboratory examinations

Real-time RT–PCR tests were performed to detect SARS-CoV-2 nucleic acid; the detailed protocols were described previously [32]. During hospitalization, serial monitoring of 30 laboratory biomarkers (Table S1) was conducted for each COVID-19 patient, and blood samples were collected at different time points when laboratory biomarkers were requested by physicians to monitor disease progression. Laboratory biomarkers, such as routine peripheral blood biomarkers, inflammatory biomarkers, coagulation biomarkers, serum biochemical biomarkers, and anti-SARS-CoV-2 IgG and IgM antibodies, were assessed during hospitalization. Peripheral blood was collected for routine blood tests using an automated hematology analyzer. Biochemical biomarkers such as albumin, alanine aminotransferase, aspartate aminotransferase, and creatinine were measured using a Roche automated clinical chemistry analyzer (Roche Diagnostics). Coagulation tests were conducted with a new coagulation analyzer, STAR Max® (Diagnostica Stago). Serum quantitative measurements of anti-SARS-CoV-2 IgM and IgG antibodies were conducted using commercial chemiluminescence kits of iFlash-SARS-CoV-2 IgM (Cat. No. C86095M) and iFlash-SARS-CoV-2 IgG (Cat. No. C86095G) from Shenzhen YHLO Biotech. Seroconversion was defined by the cutoff of IgG ≥ 10 AU/mL or IgM ≥ 10 AU/mL.

### Statistical analysis

We analyzed either the proportion of categorical variables or the median/interquartile range (IQR) of continuous variables. Chi-square tests were applied to compare proportions of categorical variables, and Fisher’s exact tests were applied within the context of limited data. Logarithmic transformation of continuous variables with all-positive data was conducted, and normality tests of continuous variables on the original scale and the log-transformed scale were performed using Shapiro-Wilk tests. We employed two-tailed t-tests for continuous variables following normal distributions, Wilcoxon rank-sum tests for non-normal continuous variables in paired groups, and Kruskal-Wallis tests for non-normal continuous variables in unpaired groups. To analyze correlations between biomarkers, Pearson’s and Spearman’s correlation coefficients were measured for normal and non-normal continuous variables, respectively. Hierarchical clustering analysis was applied to show clustering of correlating biomarkers. Cox proportional hazards models were utilized in survival analyses to assess hazard ratios (HR) indicating the effects of risk factors on survival outcomes: either death for non-survivors or hospital discharge for survivors. Baseline values of all factors were initiated in univariate survival analysis, and significant variables were subsequently used as inputs in stepwise multivariate survival analyses. Kaplan-Meier curves were built to show survival time and log-rank tests were used to compare survival distributions between different groups. The pairwise deletion approach was applied to handle missing data. All statistical analyses were descriptive, and no random sampling was conducted. For all statistical tests, two-sided tests at the level of < 0.05 were considered significant. Above statistical analyses were performed using MATLAB R2016.

## Results

### Baseline characteristics of COVID-19 patients

A cohort of 272 hospitalized patients with laboratory-confirmed COVID-19 were enrolled in this retrospective study. At hospital admission, 99 (36.4%) patients were severely ill, 139 (51.1%) were males, and 164 (60.3%) had comorbidities (Table 1). Moreover, 64.6% (64/99) of the severely ill patients were males, and most males (66%) had comorbidities. The most common symptom was fever (85.3%), followed by cough (62.1%), fatigue (37.9%), and shortness of breath (33.5%).

**Table 1:** A cohort of hospitalized patients with laboratory-confirmed COVID-19

Characteristics	Total (***N*** = 272)	Patients aged < 65 y (***N*** = 132)		Patients aged ≥ 65 y (***N*** = 140)		***P***-value*
		Moderate (***N*** = 95)	Severe (***N*** = 37)	Moderate (***N*** = 78)	Severe (***N*** = 62)	
Male gender	139 (51.1%)	36 (37.9%)	25 (67.6%)	39 (50%)	39 (63%)	0.003
Age (years)	65 (56–72)	54 (43–60)	57 (50–62)	70 (68–75)	72 (69–78)	< 0.01
**Baseline symptoms**						
Fever	232 (85.3%)	83 (87.4%)	29 (78.4%)	67 (85.9%)	53 (85.5%)	0.62
Cough	169 (62.1%)	57 (60.0%)	24 (64.9%)	49 (62.8%)	39 (62.9%)	0.95
Fatigue	103 (37.9%)	33 (34.7%)	16 (43.2%)	25 (32.1%)	29 (46.8%)	0.25
Shortness of breath	91 (33.5%)	27 (28.4%)	16 (43.2%)	21 (26.9%)	27 (43.5%)	0.07
Chills	72 (26.5%)	24 (25.3%)	10 (27.0%)	18 (23.1%)	20 (32.3%)	0.67
Diarrhea	71 (26.1%)	25 (26.3%)	9 (24.3%)	19 (24.4%)	18 (29.0%)	0.93
Myalgia	63 (23.2%)	24 (25.3%)	10 (27.0%)	15 (19.2%)	14 (22.6%)	0.74
Headache	35 (12.9%)	15 (15.8%)	5 (13.5%)	6 (7.7%)	9 (14.5%)	0.43
Nausea	30 (11.0%)	12 (12.6%)	5 (13.5%)	6 (7.7%)	7 (11.3%)	0.71
Vomit	18 (6.6%)	6 (6.3%)	2 (5.4%)	4 (5.1%)	6 (9.7%)	0.72
Abdominal pain	11 (4.0%)	5 (5.3%)	1 (2.7%)	3 (3.8%)	2 (3.2%)	0.88
Blood cough	5 (1.8%)	1 (1.1%)	0 (0.0%)	1 (1.3%)	3 (4.8%)	0.36
**Baseline comorbidity**						
Hypertension	114 (41.9%)	21 (22.1%)	16 (43.2%)	42 (53.8%)	35 (56.5%)	< 0.01
Diabetes mellitus	71 (26.1%)	18 (18.9%)	9 (24.3%)	20 (25.6%)	24 (38.7%)	0.05
Cardiovascular disease	22 (8.1%)	2 (2.1%)	1 (2.7%)	10 (12.8%)	9 (14.5%)	0.008
Cancer	13 (4.8%)	4 (4.2%)	0 (0.0%)	6 (7.7%)	3 (4.8%)	0.34
Cerebrovascular disease	10 (3.7%)	1 (1.1%)	1 (2.7%)	3 (3.8%)	5 (8.1%)	0.15
Chronic bronchitis	8 (2.9%)	2 (2.1%)	0 (0.0%)	3 (3.8%)	3 (4.8%)	0.50
Cholecystitis	3 (1.1%)	0 (0.0%)	0 (0.0%)	2 (2.6%)	1 (1.6%)	0.44
COPD^#^	2 (0.7%)	1 (1.1%)	0 (0.0%)	0 (0.0%)	1 (1.6%)	0.65
**Comorbidity count**						< 0.01
0	108 (40%)	62 (65%)	15 (40%)	18 (23%)	13 (21%)	
1	95 (35%)	18 (19%)	17 (46%)	35 (45%)	25 (40%)	
2	46 (17%)	12 (13%)	4 (11%)	17 (22%)	13 (21%)	
≥3	23 (8%)	3 (3%)	1 (3%)	8 (10%)	11 (18%)	
**Clinical outcome**						
Death	42 (15.4%)	2 (2.2%)	11 (29.7%)	1 (1.3%)	28 (45.2%)	< 0.01
Length of hospital stay (days)	21 (12–29)	17 (10–23)	19 (13–31)	23 (18–33)	25 (10–33)	< 0.01

*: *P*-values were measured by multigroup tests of variables in four patient groups (discrete variables: chi-squared tests, continuous variables: Kruskal-Wallis tests)

#: COPD: chronic obstructive pulmonary disease

**Fig. 1:**
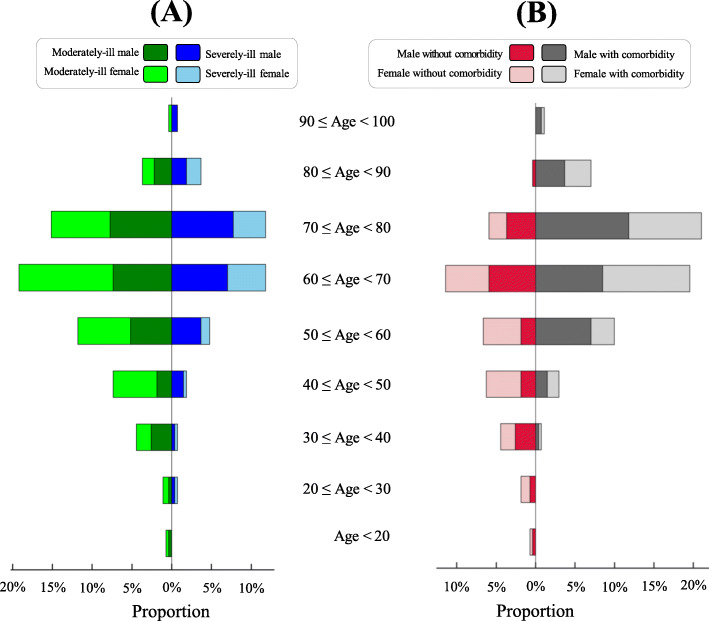
Age distribution of 272 hospitalized patients with COVID-19. **A** Proportions of COVID-19 males/females who were moderately-ill (left) or severely-ill (right) at hospital admission. **B** Proportions of COVID-19 males/females without any comorbidity (left) or with comorbidity (right) at hospital admission

Age distribution is shown in Fig. 1. The median age was 65 years (interquartile range: 56 to 72 years); the youngest patient was a 14-year-old female and the oldest a 92-year-old male. Subsequently, two patient groups were defined: age < 65 years (*N* = 132) and age ≥ 65 years (*N* = 140). The proportion of males was higher in patients aged ≥65 years than in those aged < 65 years (55.7% versus 46%, *p*-value = 0.12). Compared with patients aged < 65 years, older patients had more severe cases (44.3% versus 28%, *p*-value < 0.01) and comorbidities (77.9% versus 41.7%, *p*-value < 0.01). In patients aged ≥65 years, highly prevalent comorbidities included hypertension (55%), diabetes mellitus (31.4%), and cardiovascular disease (13.6%, Table 1).

**Fig. 2:**
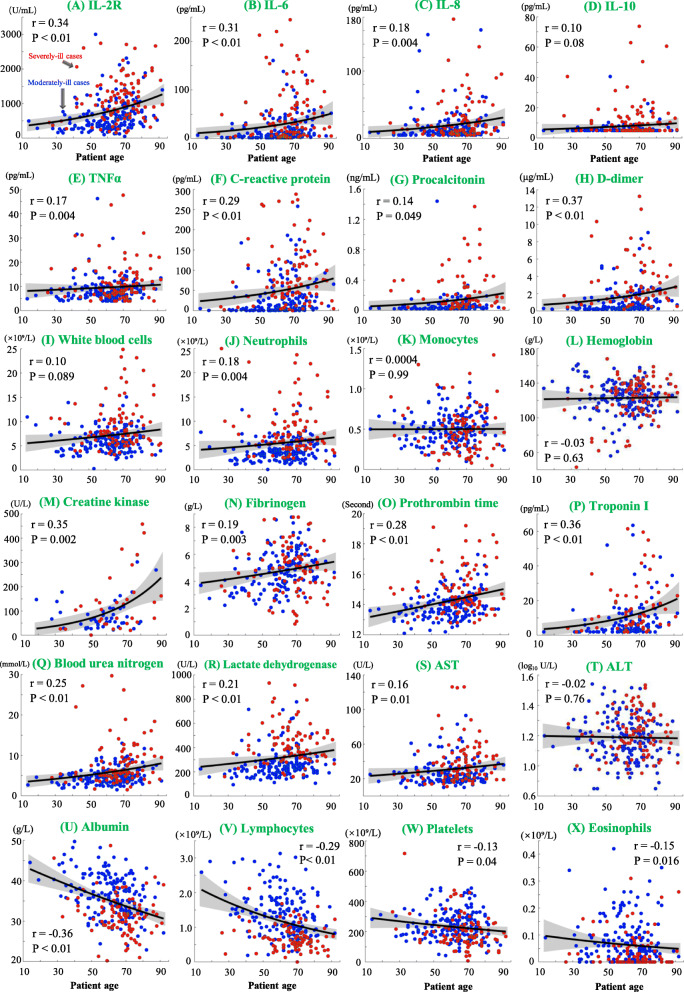
Correlation of baseline biomarkers with patient age. Red and blue dots indicate results from severely-ill and moderately-ill patients, respectively. The fitted linear polynomial curves and 95% confidence interval bounds are shown by black lines and gray area, respectively. Pearson’s correlation coefficients were measured for albumin (g/L), fibrinogen (g/L) and ALT (log_10_ U/L), while Spearman’s correlation coefficients were measured for other biomarkers whose distributions were not normal. Correlation coefficients (*r*) and their *p*-values (*P*) are shown

Baseline levels of blood biomarkers were associated with patient age in subsets of moderately ill patients and severely ill patients. As shown in Fig. 2, increasing age correlated positively with serum levels of individual biomarkers, including IL-2R, IL-6, C-reactive protein, D-dimer, creatine kinase, and troponin I (*p*-values < 0.05). In contrast, patient age correlated negatively with the decreasing levels of biomarkers, including albumin, lymphocytes, eosinophils, and platelets (*p*-values < 0.05).

### Dynamics of cytokines and blood biomarkers during disease progression

To monitor disease progression, 1769 blood samples were collected from 272 COVID-19 patients to measure the serum levels of cytokines (IL-2R, IL-6, IL-8, IL-10, TNF-α) and blood biomarkers during the hospitalization. Blood samples were collected at the time of physician requests (usually once per week). Because patient age plus disease severity may play a role in biomarker dynamics, our subsequent analyses focused on the weekly dynamics of the above biomarkers in four subgroups: (i) moderately ill patients aged < 65 years, (ii) severely ill patients aged < 65 years, (iii) moderately ill patients aged ≥65 years, and (iv) severely ill patients aged ≥65 years.

**Fig. 3:**
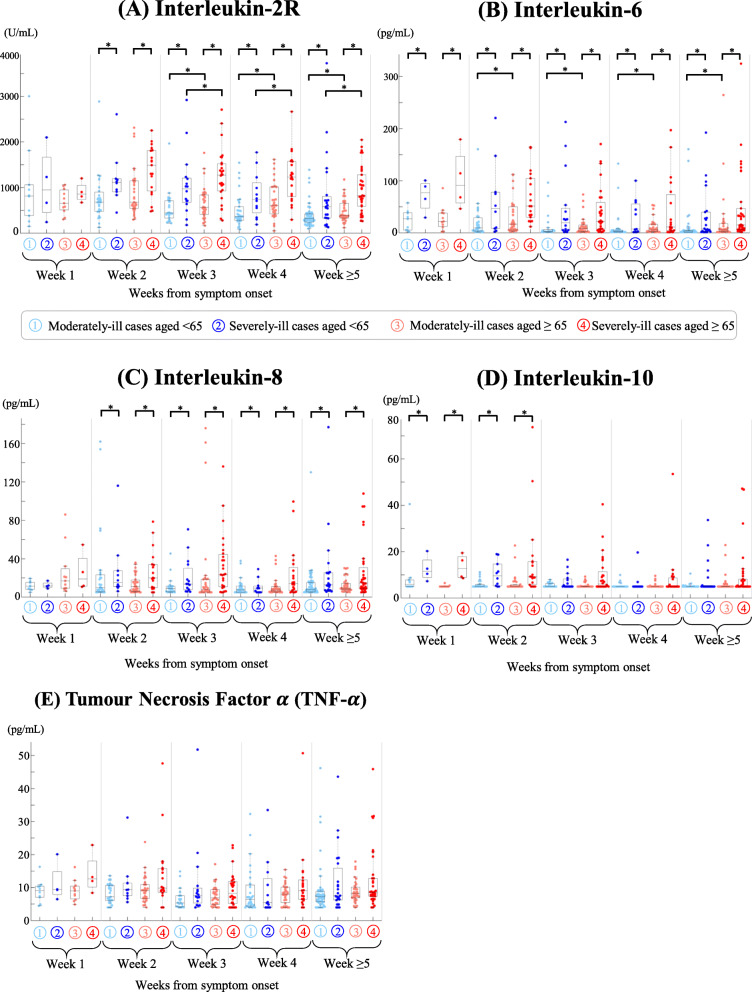
Dynamics of five cytokines (IL-2R, IL-6, IL-8, IL-10, TNF-α) in COVID-19 patients during hospitalization. Four patient groups were visualized in light blue (moderately-ill patients aged < 65 years), blue (severely-ill patient aged < 65 years), light red (moderately-ill patients aged ≥65 years), and red (severely-ill patient aged ≥65 years). Due to their non-normality, cytokine levels were compared using Wilcoxon rank-sum tests. A significant difference (*p*-value < 0.05) was highlighted by an asterisk *. One sample from each patient was obtained from week 1, week 2, week 3, week 4 to week ≥5 after symptom onset. If one patient had multiple tests within one week, median results were obtained for our analysis

Serum levels of IL-2R, IL-6, IL-8, and IL-10 were higher in severely-ill patients than in moderately ill patients. Significant differences in IL-2R (week 2 to week ≥5**,** Fig. 3A), IL-6 (week 1 to week ≥5**,** Fig. 3B), IL-8 (week 2 to week ≥5**,** Fig. 3C), and IL-10 (week 1 to week 2, Fig. 3D) were observed in the comparison of moderately ill versus severely ill patients aged < 65 as well as moderately ill versus severely ill patients aged ≥65 years. Serum levels of TNF-α were slightly higher in severely ill patients, but no significant difference was found within the context of COVID-19 severity and patient age (Fig. 3E).

Analyses of 24 non-cytokine biomarkers revealed their possible associations with disease severity and patient age (Fig. S1-S4). Among these biomarkers, serum levels of six biomarkers (D-dimer, C-reactive protein, procalcitonin, lymphocytes, neutrophils, albumin) consistently showed increasing or decreasing patterns in the four patient subgroups (Fig. S1).

### IgG and IgM dynamics during disease progression

Serum levels of anti-SARS-CoV-2 IgG and IgM antibodies were measured in a subcohort of 162 patients with COVID-19. The earliest date and the latest date of IgG/IgM tests was 10 days and 76 days after symptom onset, respectively. Note that all blood samples were collected during the hospitalization, such that IgG/IgM tests were not conducted before hospital admission or after hospital discharge.

**Fig. 4:**
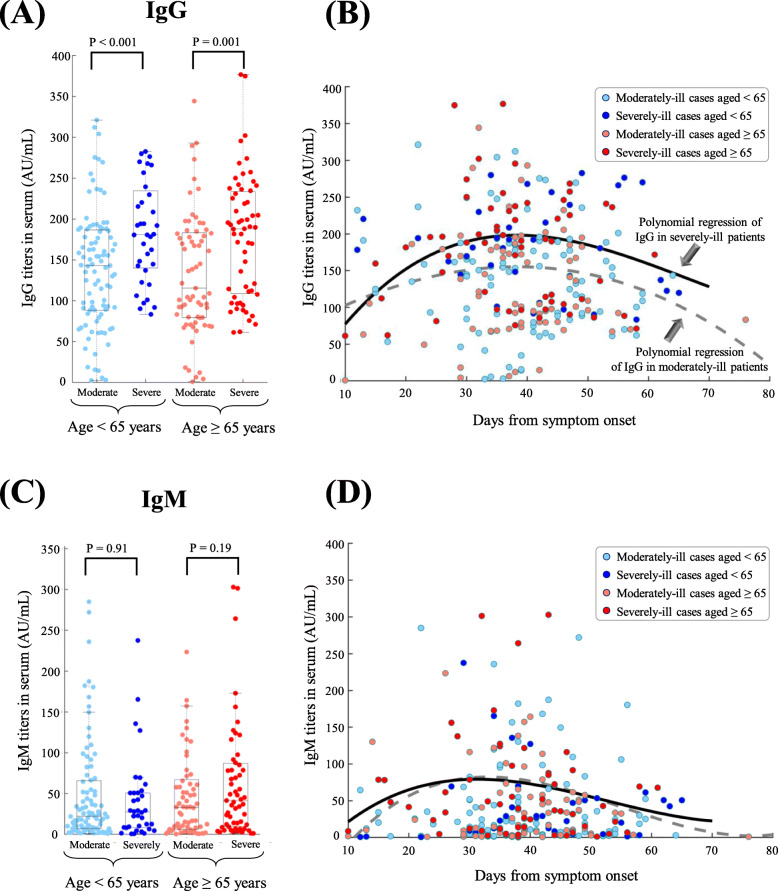
Serum levels of anti-SARS-CoV-2 IgG and IgM antibodies. **A** IgG levels in four patient groups: moderately-ill patients aged < 65 years (light blue), severely-ill patients aged < 65 years (blue), moderately-ill patients aged ≥65 years (light red), and severely-ill patients aged ≥65 years (red). IgG data was collected across all timepoints. **B** Polynomial regression of IgG was applied to fit the dynamic patterns of IgG from symptom onset to clinical outcomes (hospital discharge or death). **C** IgM levels in four patient groups. **D** Polynomial regression of IgM dynamics from symptom onset to clinical outcomes

Based on the collection of blood samples during hospitalization, IgG titers were higher in severely ill patients than in moderately ill patients (median: 187.7 versus 137.5 AU/mL, *p*-value < 0.01, Fig. 4A). The dynamics of IgG titers changed during disease progression, and with a steady IgG increase in the first 4 weeks (Fig. 4B). From week 5 to week 10, a decline in IgG titers was observed in moderately ill patients, whereas severely ill patients maintained relatively higher levels of IgG titers even at week 10 (Fig. 4B). Comparisons of IgM titers showed no significant difference with regard to patient age and COVID-19 severity (Fig. 4C). A similar pattern of IgM dynamics was observed in both moderately-ill and severely-ill patients, though IgM was almost undetectable at week 10 (Fig. 4D). In total, 86.7% of survivors and 100% of non-survivors experienced IgG seroconversion (IgG ≥10 AU/mL) and/or IgM seroconversion (IgM ≥10 AU/mL).

### Anti-SARS-CoV-2 IgG responses are associated with early-stage cytokines

**Fig. 5:**
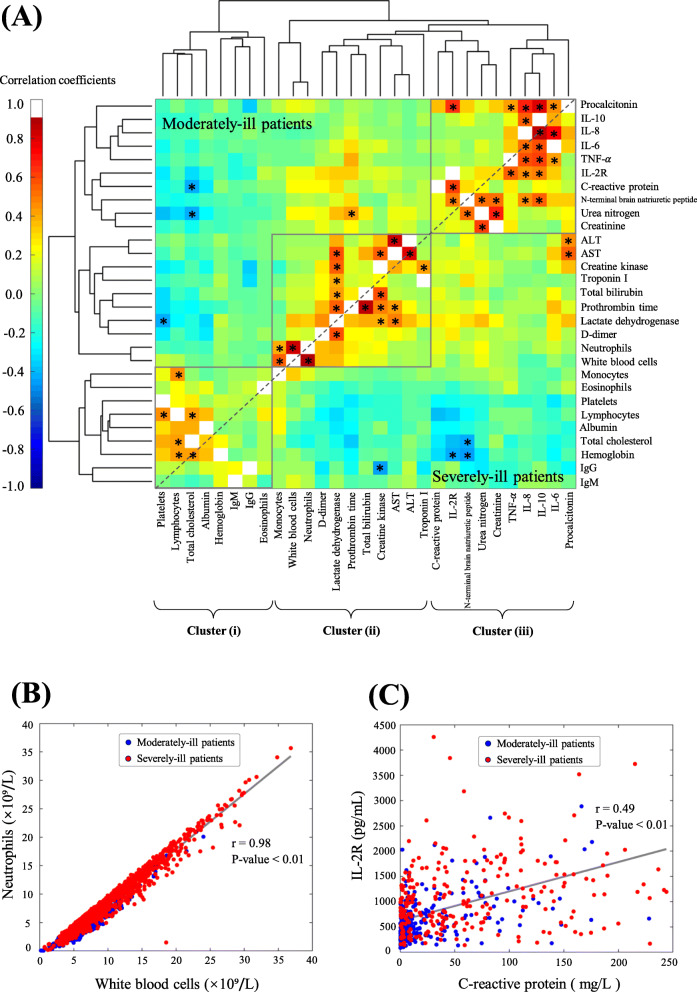
Clustering analyses of biological biomarkers in COVID-19 patients. **A** Hierarchical clustering of 29 biological biomarkers based on the subsets of moderately-ill patients (top left) and severely-ill patients (bottom right). The significance of any correlation coefficient (absolute value > 0.4 and *p*-value < 0.05) was highlighted by an asterisk *. Three clusters (i), (ii), and (iii) were indicated based on the hierarchical clustering. **B** Plots of white blood cells versus neutrophils. **C** Plots of C-reactive protein versus IL-2R

Given that many blood biomarkers are assessed in clinical diagnosis, we determined whether these biomarkers correlate with each other within the context of COVID-19 severity. Pairwise correlation coefficients were measured based on all available data for moderately ill and severely-ill patients (Fig. 5A). The highest correlation coefficient (r = 0.98, *p*-value < 0.01) was identified between white blood cells and neutrophils (Fig. 5B), and this is confirmed by the fact that neutrophils represent the most plentiful type of white blood cells. Clustering analyses further revealed three major clusters of biomarkers: (i) IgG, IgM, eosinophils, platelets, lymphocytes, albumin, total cholesterol, and hemoglobin; (ii) AST, ALT, D-dimer, lactate dehydrogenase, prothrombin time, total bilirubin, troponin I, and creatine kinase; and (iii) IL-2R, IL-6, IL-8, IL-10, TNF-α, procalcitonin, creatinine, C-reactive protein, N-terminal brain natriuretic peptide, creatinine, and urea nitrogen (Fig. 5A). In cluster (i), IgG and IgM were closely correlated in the same branch. In cluster (ii), known biomarkers such as white blood cells and neutrophils were clustered. Four cytokines (IL-6, IL-8, IL-10, and TNF-α) were clustered in the cluster (iii). IL-2R correlated positively with C-reactive protein (Fig. 5C), a biomarker known for its response to infection and/or inflammation.

**Fig. 6:**
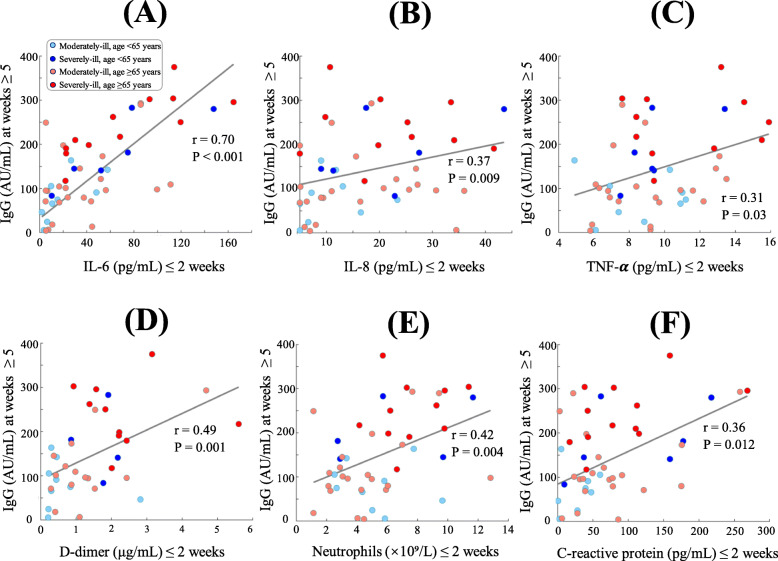
Scatter plots of early-stage biomarkers (≤ 2 weeks from symptom onset) versus late-stage IgG (≥ 5 weeks from symptom onset) in COVID-19 patients. Serum levels of cytokines IL-6 (**A**), IL-8 (**B**), TNF-α (**C**) at the early stage of ≤2 weeks from symptom onset are positively correlated with the response of IgG at the late stage of ≥5 weeks from symptom onset. Positive correlations with IgG responses were also observed for D-dimer (**D**), neutrophils (**E**), and C-reactive protein (**F**). Spearman’s correlation coefficients (*r*) and their *p*-values (*P*) are shown

We next asked whether late-stage IgG responses (from week 5 to 10) are associated with early-stage biomarker responses (≤ 2 weeks) in COVID-19 patients. To this end, we analyzed a subcohort of 47 hospitalized patients whose blood samples were tested within two weeks after symptom onset and whose late-stage IgG titers were also tested from weeks 5 to 10. Among all available biomarkers, early responses of six biomarkers (IL-6, IL-8, TNF-α, D-dimer, neutrophils, C-reactive protein) correlated significantly with high titers of anti-SARS-CoV-2 IgG antibodies (*p*-values < 0.05) (Fig. 6). Of note, the pairwise correlation coefficient between early-stage IL-6 and late-stage IgG was 0.70 (*p*-value < 0.001, Fig. 6A) and a linear regression model could be built as Y = 71.62 + 1.62X (R^2^: 49.26%) to predict late-stage IgG responses (the variable Y) based on early-stage IL-6 levels (the variable X). This significant correlation was independently observed in the subsets of moderately-ill patients (r = 0.62, *p*-value < 0.001) and severely-ill patients (r = 0.83, *p*-value < 0.001). Due to a steady decrease in IgM titers from ≥5 weeks, significant correlations of IgG titers with early-stage biomarkers were not observed.

### Risk factors associated with the mortality of COVID-19 patients

In our cohort of 272 hospitalized patients, 42 (15.4%) died, and the median length of hospital stay was 21 days (Table 1). Deaths mostly occurred in severely ill patients, both aged ≥65 years (45.9%) and < 65 years (31.4%). In contrast, few deaths were observed in moderately-ill patients aged < 65 years (2.2%) and ≥ 65 years (1.3%). Moreover, the median length of hospital stay was longer in patients aged ≥65 than in those aged < 65 years (23 versus 18 days, *p*-value = 0.0003).

Using 62 baseline parameters (patient age, gender, baseline disease severity, symptoms, comorbidities, blood biomarkers) as predictors, Cox proportional hazards models were applied to analyze risk factors potentially associated with the clinical outcome of either death for non-survivors or hospital discharge for survivors (Table 2). Univariate survival analyses identified 24 of 62 baseline parameters as risk factors, such as male sex, patient age, and baseline severity (*p*-values < 0.05, Table 2). These 24 risk factors were used as inputs in multivariate survival analyses, which further identified four risk factors: patient age (hazard ratio (HR): 1.07, *p*-value = 0.002), baseline severity (HR: 33.7, *p*-value = 0.0009), baseline D-dimer (HR: 1.09, *p*-value = 0.008), and baseline IL-6 (HR: 1.12, *p*-value = 0.025).

**Table 2:** Risk factors associated with the clinical outcome (death or hospital discharge) of COVID-19 patients using Cox proportional hazards models

	Univariate analysis*		Multivariate analysis	
	HR (95% CI)	*P*-value	HR (95% CI)	*P*-value
Male gender	2.23 (1.60 to 3.12)	0.016		
Patient age ^&^	1.05 (1.03 to 1.06)	9.8 × 10^−4^	1.07 (1.04 to 1.09)	0.002
Baseline disease severity	24.2 (13.3 to 44.1)	1.1 × 10^−7^	38.0 (12.9 to 111.3)	7 × 10^− 4^
**Baseline symptoms**				
Fatigue	2.02 (1.48 to 2.75)	0.023		
**Baseline comorbidity**				
Cardiovascular disease	2.33 (1.54 to 3.53)	0.041		
**Blood routine biomarkers**				
White blood cells	1.18 (1.15 to 1.21)	3 × 10^−10^		
Neutrophils	1.14 (1.11 to 1.16)	2.2 × 10^−9^		
Lymphocytes	0.03 (0.02 to 0.06)	6 × 10^− 10^		
**Coagulation biomarkers**				
D-dimer	1.10 (1.08 to 1.12)	5.4 × 10^−8^	1.09 (1.06 to 1.13)	0.006
Prothrombin time	1.57 (1.43 to 1.72)	9.6 × 10^−7^		
**Biochemistry biomarkers**				
Albumin	0.85 (0.82 to 0.87)	4.8 × 10^−4^		
Aspartate aminotransferase	1.02 (1.01 to 1.03)	6.2 × 10^− 4^		
Total cholesterol	0.56 (0.46 to 0.67)	0.0017		
Total bilirubin	1.08 (1.06 to 1.11)	0.0001		
Blood urea nitrogen	1.15 (1.12 to 1.17)	8.3 × 10^−3^		
Creatinine	1.02 (1.01 to 1.02)	6.3 × 10^−5^		
**Cardiac biomarkers**				
Troponin I	1.03 (1.02 to 1.03)	5 × 10^−3^		
Myocardial creatine kinase	1.38 (1.29 to 1.49)	7.6 × 10^−6^		
**Inflammatory biomarkers**				
C-reactive protein	1.01 (1.01 to 1.01)	5.9 × 10^−9^		
Procalcitonin	8.38 (5.56 to 12.6)	2.2 × 10^−7^		
IL-6	1.11 (1.10 to 1.13)	3 × 10^−14^	1.12 (1.10 to 1.14)	0.025
IL-8	1.02 (1.01 to 1.02)	6.2 × 10^−7^		
IL-10	1.03 (1.02 to 1.04)	2.7 × 10^−4^		
TNF-α	1.04 (1.03 to 1.06)	6.6 × 10^−3^		

*: A total of 62 biomarkers were tested in our survival analysis and only variables with a significant hazard ratio (HR) at the significance level of *p*-value < 0.05 are listed in the table

&: Increase per year

**Fig. 7:**
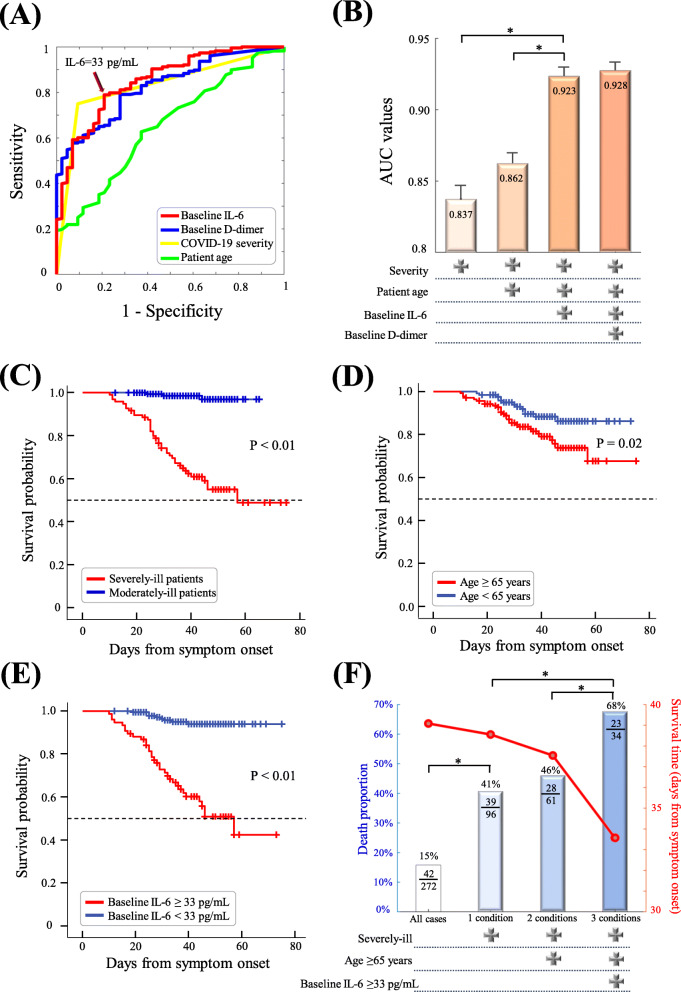
Survival analysis and area under the curves of risk factors. **A** Area under the curves (AUC) of baseline factors in the prediction of COVID-19 mortality. The cutoff of IL-6 at 33 pg/mL (sensitivity: 79%, specificity: 81%) was optimized by the highest point of AUC based on the Cutoff Finder tool (https://molpathoheidelberg.shinyapps.io/CutoffFinder_v1/). **B** AUC values in the stepwise combination of four baseline factors. Kaplan-Meier curves are shown for severely-ill versus moderately-ill patients **(C)**, patients aged < 65 versus ≥65 years **(D)**, patients with baseline IL-6 ≥ 33 pg/mL versus patients with IL-6 < 33 pg/mL **(E)**. **F** Mortality rate and survival time of patients with the combined conditions of severely-ill, age ≥ 65, and baseline IL-6 ≥ 33 pg/mL

In predicting COVID-19 mortality, Fig. 7A shows the area under the curve for each individual factor, including patient age (AUC: 0.65, *p*-value = 0.001), baseline severity (AUC: 0.837, *p*-value < 0.01), baseline D-dimer (AUC: 0.827, *p*-value < 0.01), and baseline IL-6 (AUC: 0.856, *p*-value < 0.01). Of note, the area under the curve for three factors (patient age, baseline severity, baseline IL-6) reached 0.923 (Fig. 7B). Kaplan-Meier curves were subsequently evaluated to examine whether these three risk factors affect the survival time from symptom onset. The mean survival time was significantly different in comparisons of severely-ill versus moderately-ill patients (*p*-value < 0.01, Fig. 7C), patients aged ≥65 versus < 65 (*p*-value =0.02, Fig. 7D), and patients with baseline IL-6 ≥ 33 pg/mL versus IL-6 < 33 pg/mL (*p*-value < 0.01, Fig. 7E). Compared with the overall cohort, the mortality rate was significantly higher in the subgroup of patients with these risk factors, especially severely-ill old patients with IL-6 ≥ 33 pg/mL at baseline (Fig. 7F).

## Discussion

At the early stage, invasion of SARS-CoV-2 leads to activation of innate immunity and dendritic cells with excessive release of cytokines (e.g., IL-6, TNF-α) and chemokines (e.g., CCL20, CXCL1, CXCL2), thereafter driving induction of SARS-CoV-2-specific B cell and T cell responses [33]. Subsequently, plasma B cells are responsible for production of anti-SARS-CoV-2 IgG and IgM antibodies that act in adaptive immune responses [2, 3]. Our study was initiated to test the hypothesis of whether early responses of cytokines are associated with late-stage responses of anti-SARS-CoV-2 IgG/IgM antibodies. As of January 2022, the majority of the populations has been vaccinated (> 90% in China), and many cytokine-targeted drugs (e.g., tocilizumab [34]) are widely used in clinical practice [35]. For this reason, we decided to perform a retrospective study to collect detailed cytokine and antibody records for hospitalized COVID-19 patients during the early outbreak of the pandemic when vaccines and cytokine-targeted drugs were not available or administered. Based on detailed records of hospitalized patients, our findings can be briefly summarized, as indicated below. (i) Serum levels of certain cytokines and antibodies are associated with patient age and COVID-19 severity at baseline. Older patients with severe COVID-19 usually have higher levels of serum IL-6 and IgG antibodies. (ii) Baseline factors such as patient age, disease severity, and IL-6 are early predictors of clinical outcomes among COVID-19 patients. (iii) Early-stage cytokines such as IL-6 correlate positively with late-stage IgG responses, especially in older patients.

In agreement with previous studies [36–38], our analyses of cytokine profiles revealed different dynamics of IL-2R, IL-6, IL-8, IL-10, and TNF-α during the disease progression of COVID-19. We observed that serum IL-6 exhibited an early response to disease severity even at the first week after symptom onset, supporting its role as an early predictor of severe disease (Fig. 3B). This is in agreement with previous studies that showed IL-6 to be a sensitive and specific predictor of disease severity [19, 39] and clinical outcomes such as death and respiratory failure [40–42]. Moreover, we observed that IL-6 and IL-2R were associated with older age and disease severity (Fig. 3A, B). A meta-analysis also reported that older age might significantly influence the association of IL-6 with clinical outcomes [43]. Such age-severity association indicates the existence of age-associated immune signatures and the need for better clinical management of severely-ill older patients [44]. In addition to IL-6 and IL-2R, we observed associations of serum IL-8 and IL-10 with disease severity, which is in agreement with previous studies [19, 37, 45]. Our univariate survival analyses suggested that IL-6, IL-8, IL-10, and interferon-α are risk factors significantly associated with the clinical outcome of death; IL-6 remained a key factor in the multivariate survival model (Table 2). The role of IL-6 in the disease progression of COVID-19 has been widely recognized [46], and IL-6 blockade possibly reduce the systemic inflammation [47]. Although an IL-6 inhibitor called tocilizumab offered a modest reduction in mortality (31% vs. 35%) in the RECOVERY trial [48], other clinical trials (e.g., the REMAP-CAP trial) showed no benefit of IL-6 inhibition in reducing mortality [47, 49]. Future studies are needed to address whether antagonists of IL-6 and its receptor would be beneficial for better clinical outcomes.

Responses to anti-SARS-CoV-2 antibodies are associated with many baseline factors such as COVID-19 severity, patient age, and baseline symptoms [50–53]. Our analyses showed a steady increase in anti-SARS-CoV-2 IgG titers in the first month after symptom onset, and severely ill patients maintained relatively high levels of IgG even at week 10 (Fig. 4). In contrast, IgM peaked at nearly 5 weeks after symptom onset and almost disappeared by week 10. In agreement with previous results, IgM antibodies, accounting for approximately 10% of human immunoglobulins, are often produced during the early phase of acute infection [50]. IgG antibodies, accounting for approximately 75% of human immunoglobulins, provide long-term immunity after viral infection [50]. In an Italian study of asymptomatic to critically ill patients, IgM seroconversion disappeared at 4 months and 47% of IgG seroconversion was observed at 10 months after symptom onset [51]. A Brazilian study reported a 30.4% loss of IgG reactivity after 90 days of symptom onset [52]. A Chinese study also showed the maintenance of IgG responses even up to 10 months after infection [53]. In agreement with our findings, a previous study observed higher titers of IgG antibodies in severely ill older patients, and the antibody decline was associated with antibody titers and symptoms at baseline [51].

Age-related features of COVID-19 cytokine profiles and immune responses have been observed by our study and in other studies [54–56]. We detected different levels of serum IL-6, IL-2R, albumin, D-dimer, and C-reactive protein between young and older patients (Fig. 3, Fig. S1). Patient age was also a key risk factor in our survival analysis (Table 2). Ageing is associated with elevated systemic levels of inflammatory cytokines (e.g., IL-6, IL-8, TNF-α, IL-13, interferon-γ) as well as acute-phase proteins [57]. Older patients ≥65 years may experience impaired adaptive responses with the scarcity of naïve T cells, therefore failing to activate antigen-specific responses to control COVID-19 [55]. Older patients may experience immunosenescence with ineffective viral responses to fight COVID-19 due to age-related biological changes (e.g., impaired innate immune responses), chronic disease states (e.g., cardiovascular disease, diabetes), and environmental risk factors (e.g., smoking) [9, 54]. In addition to patient age and cytokines, other factors, such as sex [58, 59] and lymphocytes [10], might be associated with the disease outcomes of COVID-19 patients. The mortality risk of COVID-19 was often higher in males than females [59]. Serum levels of CD8+ T lymphocytes were significantly lower in severe cases than in mild and moderate cases with COVID-19 [10]. Moreover, a severity-associated decrease in CD4+ T lymphocytes was observed in males with COVID-19 [10].

There are several limitations to this study. First, this retrospective study analyzed a cohort of COVID-19 patients hospitalized during the early pandemic to avoid the potential impact of different vaccines, cytokine antagonists, and SARS-CoV-2 variants. Although our study sought to analyze immune responses of different variants (e.g., omicron, alpha, beta, gamma, delta) [60], > 90% of citizens in Wuhan have been vaccinated, and only a few cases (*N* < 100 in total) have been reported after December 2020 because of the strict adherence to a zero-COVID-19 policy in China. Second, our study reported the interplay between early-stage cytokines and late-stage IgG due to active immune responses, but any causal effect remains unclear and future studies need to address the exact molecular mechanisms. Third, blood samples in our retrospective study were not collected prior to SARS-CoV-2 infection due to the sudden outbreak in early 2020. Future studies should address the baseline impact of inflamm-ageing in older patients with COVID-19. Fourth, more than 10 cytokines/chemokines are possibly involved in the disease progression of COVID-19, but our study was limited to those biomarkers that had been tested in our hospital laboratory. It is worth examining a full panel of cytokines/chemokines in future studies. Whether cytokine antagonists reduce the risk of cytokine storms and improve the clinical outcomes of different variants should also be addressed.

## Conclusion

Based on a cohort of hospitalized COVID-19 patients with detailed records of cytokines, antibodies, and blood biomarkers, this retrospective study supports the hypothesis that early responses of elevated cytokines such as IL-6 may reflect active responses of the humoral immune system (Fig. S5). Active immune responses drive production of high IgG titers at the late stage, ideally delivering long-term protection. IL-6 can be used as an early predictor of IgG responses and cytokine storms, which may cause fatal symptoms such as acute respiratory distress syndrome. With a limited number of antiviral therapies against COVID-19 [61–64], future studies need to investigate whether antagonists of IL-6 and its receptor significantly improve the clinical outcomes of COVID-19.

## Supplementary Information

**Additional file 1: Table S1.** Summary of laboratory-confirmed biomarkers in our study. **Fig. S1.** Dynamics of albumin (**A**), D-dimer (**B**), lymphocytes (**C**), C-reactive protein (**D**), neutrophils (**E**), procalcitonin (**F**) in COVID-19 patients. Four groups were visualized in light blue (moderately-ill patients aged < 65 years), blue (severely-ill patients aged < 65 years), light red (moderately-ill patients aged ≥65 years), and red (severely-ill patients aged ≥65 years). **Fig. S2.** Dynamics of creatine (**A**), creatine kinase (**B**), prothrombin time (**C**), total cholesterol (**D**), urea nitrogen (**E**), total bilirubin (**F**), troponin I (**G**), N-terminal brain natriuretic peptide (**H**) in COVID-19 patients. Four groups were visualized in light blue (moderately-ill patients aged < 65 years), blue (severely-ill patients aged < 65 years), light red (moderately-ill patients aged ≥65 years), and red (severely-ill patients aged ≥65 years). **Fig. S3.** Dynamics of monocytes (**A**), platelets (**B**), eosinophils (**C**), white blood cells (**D**), hemoglobin (**E**), and fibrinogen (**F**) in COVID-19 patients. Four patient groups were visualized in light blue (moderately-ill patients aged < 65 years), blue (severely-ill patients aged < 65 years), light red (moderately-ill patients aged ≥65 years), and red (severely-ill patients aged ≥65 years). **Fig. S4.** Dynamics of aspartate aminotransferase (**A**), alanine aminotransferase (**B**), lactate dehydrogenase (**C**), and erythrocyte sedimentation rate (**D**) in COVID-19 patients. Four patient groups were visualized in light blue (moderately-ill patients aged < 65 years), blue (severely-ill patients aged < 65 years), light red (moderately-ill patients aged ≥65 years), and red (severely-ill patients aged ≥65 years). **Fig. S5.** Our hypothesis of the cytokine - antibody associations during the disease progression of COVID-19. This figure is adapted from previous publications. Briefly, the disease progression of SARS-CoV-2 can be intuitively divided into the early stage (nearly 2 weeks after symptom onset) and the late stage (> 2 weeks after symptom onset). During the early stage, SARS-CoV-2 infects airway epithelial cells with the surface receptors such as ACE2 and TMPRSS2. The active replication and release of viral particles cause host cells to trigger the generation of pro-inflammatory cytokines (e.g., IL-6, IL-10, TNF), chemokines (e.g., CCL2, CCL3, CCL5, CXCL10), and interferons (type I/III interferons). This attracts monocytes, macrophages, and T cells to the infected cells and establish a pro-inflammatory feedback loop. The innate immunity responses to SARS-CoV-2 by activating many signaling pathways during the early stage of infection, while the adaptive immune responses take over during the late stage with production of antibodies such as anti-SARS-CoV-2 IgG. The defective immune response causes the overproduction of cytokines, resulting in cytokine storm that causes fatal symptoms such as acute respiratory distress syndrome (ARDS), severe pneumonia, multiorgan failure, and coagulation damage. The red lines indicate findings of our study that early responses of cytokines are associated with IgG responses at the late stage, and baseline cytokines and other factors such as older age can be early predictors of death outcome.

## Data Availability

All data are contained within the article and available upon request after the approval of proposals by the Ethics Committees of The Second Xiangya Hospital.

## Abbreviations

ALT: alanine aminotransferase
ARDS: acute respiratory distress syndrome
AST: aspartate aminotransferase
AUC: area under the curve
CCL20: C-C motif chemokine ligand 20
COPD: chronic obstructive pulmonary disease
COVID-19: coronavirus disease 2019
CXCL: C-X-C motif chemokine receptor
HR: hazard ratios
IgG: immunoglobulin G
IgM: immunoglobulin M
IL: interleukin
IL-2R: interleukin-2 receptor
IQR: interquartile range
RT-PCR: reverse transcription polymerase chain reaction
SARS-CoV-2: severe acute respiratory syndrome coronavirus 2
TNF-α: tumor necrosis factor-alpha
