# Supplementary material for: Association between inflammatory cytokines and anti-SARS-CoV-2 antibodies in hospitalized patients with COVID-19
Source: Immun Ageing. 2022 Mar 5;19:12. doi: 10.1186/s12979-022-00271-2 (PMC8897556; doi:10.1186/s12979-022-00271-2)
Supplement: Supplementary file 1 — Additional file 1: Table S1. Summary of laboratory-confirmed biomarkers in our study. Fig. S1. Dynamics of albumin (A), D-dimer (B), lymphocytes (C), C-reactive protein (D), neutrophils (E), procalcitonin (F) in COVID-19 patients. Four groups were visualized in light blue (moderately-ill patients aged < 65 years), blue (severely-ill patients aged < 65 years), light red (moderately-ill patients aged ≥65 years), and red (severely-ill patients aged ≥65 years). Fig. S2. Dynamics of creatine (A), creatine kinase (B), prothrombin time (C), total cholesterol (D), urea nitrogen (E), total bilirubin (F), troponin I (G), N-terminal brain natriuretic peptide (H) in COVID-19 patients. Four groups were visualized in light blue (moderately-ill patients aged < 65 years), blue (severely-ill patients aged < 65 years), light red (moderately-ill patients aged ≥65 years), and red (severely-ill patients aged ≥65 years). Fig. S3. Dynamics of monocytes (A), platelets (B), eosinophils (C), white blood cells (D), hemoglobin (E), and fibrinogen (F) in COVID-19 patients. Four patient groups were visualized in light blue (moderately-ill patients aged < 65 years), blue (severely-ill patients aged < 65 years), light red (moderately-ill patients aged ≥65 years), and red (severely-ill patients aged ≥65 years). Fig. S4. Dynamics of aspartate aminotransferase (A), alanine aminotransferase (B), lactate dehydrogenase (C), and erythrocyte sedimentation rate (D) in COVID-19 patients. Four patient groups were visualized in light blue (moderately-ill patients aged < 65 years), blue (severely-ill patients aged < 65 years), light red (moderately-ill patients aged ≥65 years), and red (severely-ill patients aged ≥65 years). Fig. S5. Our hypothesis of the cytokine - antibody associations during the disease progression of COVID-19. This figure is adapted from previous publications. Briefly, the disease progression of SARS-CoV-2 can be intuitively divided into the early stage (nearly 2 weeks after symptom onset) [file 12979_2022_271_MOESM1_ESM.pdf]

Supporting Information

Table S1: Summary of laboratory-confirmed biomarkers in our study

**Blood routine biomarkers**	White blood cells (×10^9 /L)<br>Neutrophils (×10^9 /L)<br>Lymphocytes (×10^9 /L)<br>Eosinophils (×10^9 /L)<br>Monocytes (×10^9 /L)<br>Platelets (×10^9 /L)<br>Hemoglobin (g/L)
**Coagulation biomarkers**	D-dimer (μg/mL)<br>Prothrombin time (s)<br>Fibrinogen (g/L)
**Blood biochemistry biomarkers**	Alanine aminotransferase (U/L)<br>Aspartate aminotransferase (U/L)<br>Total bilirubin (μmol/L)<br>Albumin (g/L)<br>Creatinine (μmol/L)<br>Creatine kinase (U/L)<br>Lactic dehydrogenase (U/L)<br>Blood urea nitrogen (mmol/L)
**Cardiac biomarkers**	Troponin I (pg/mL)<br>Myocardial creatine kinase (ng/mL)<br>Brain natriuretic peptide (pg/mL)<br>Total cholesterol (mmol/L)
**Inflammatory biomarkers**	C-reactive protein (mg/L)<br>Erythrocyte sedimentation rate (mm/hour)<br>Procalcitonin (ng/mL)<br>Interleukin 2 receptor (U/mL)<br>Interleukin-6 (pg/mL)<br>Interleukin-8 (pg/mL)<br>Interleukin-10 (pg/mL)<br>Tumor necrosis factor-α (pg/mL)

Supporting Figures

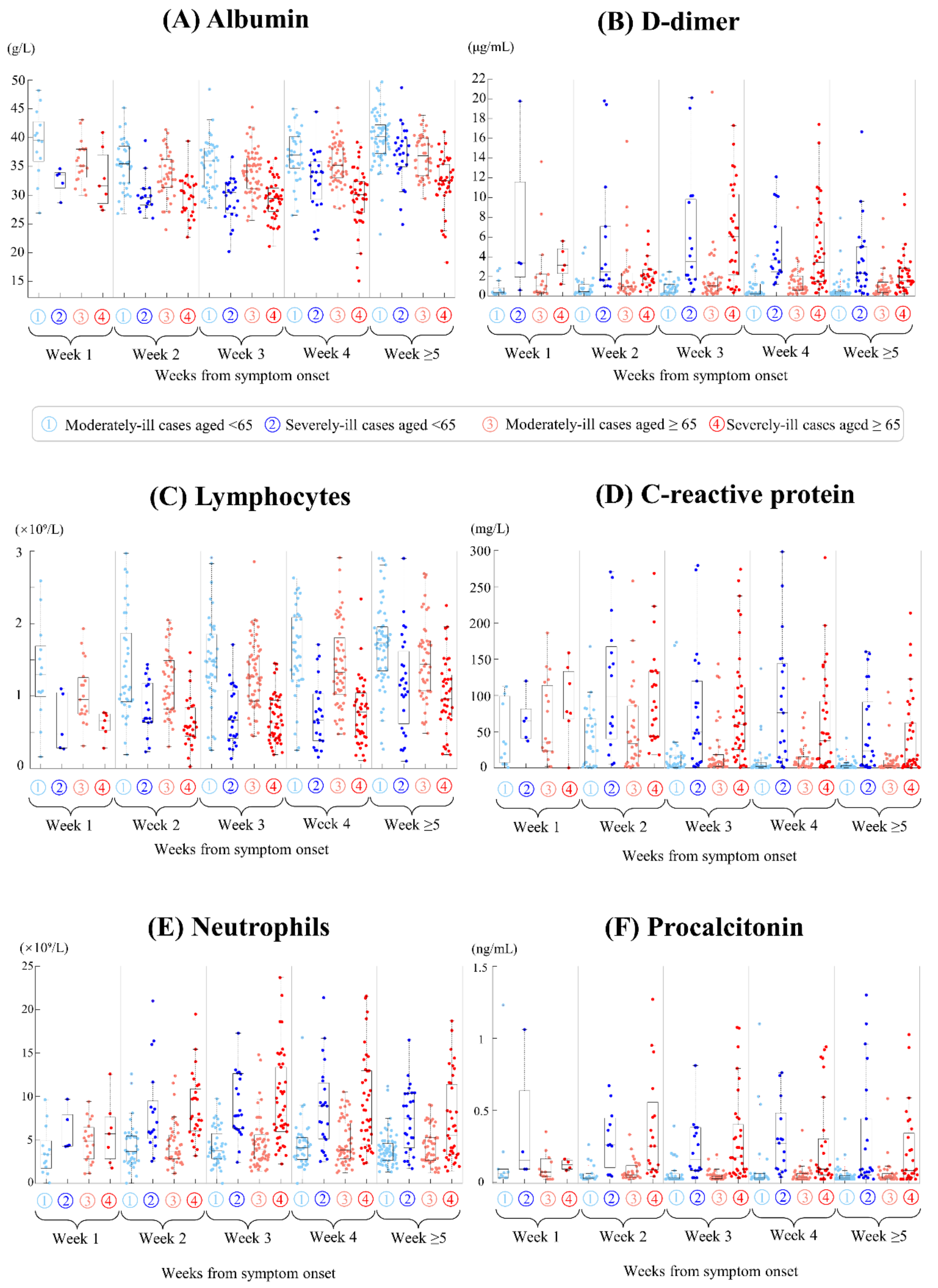

Figure S1: Dynamics of albumin (A), D-dimer (B), lymphocytes (C), C-reactive protein (D), neutrophils (E), procalcitonin (F) in COVID-19 patients. Four groups were visualized in light blue (moderately-ill patients aged <65 years), blue (severely-ill patients aged <65 years), light red (moderately-ill patients aged ≥65 years), and red (severely-ill patients aged ≥65 years).

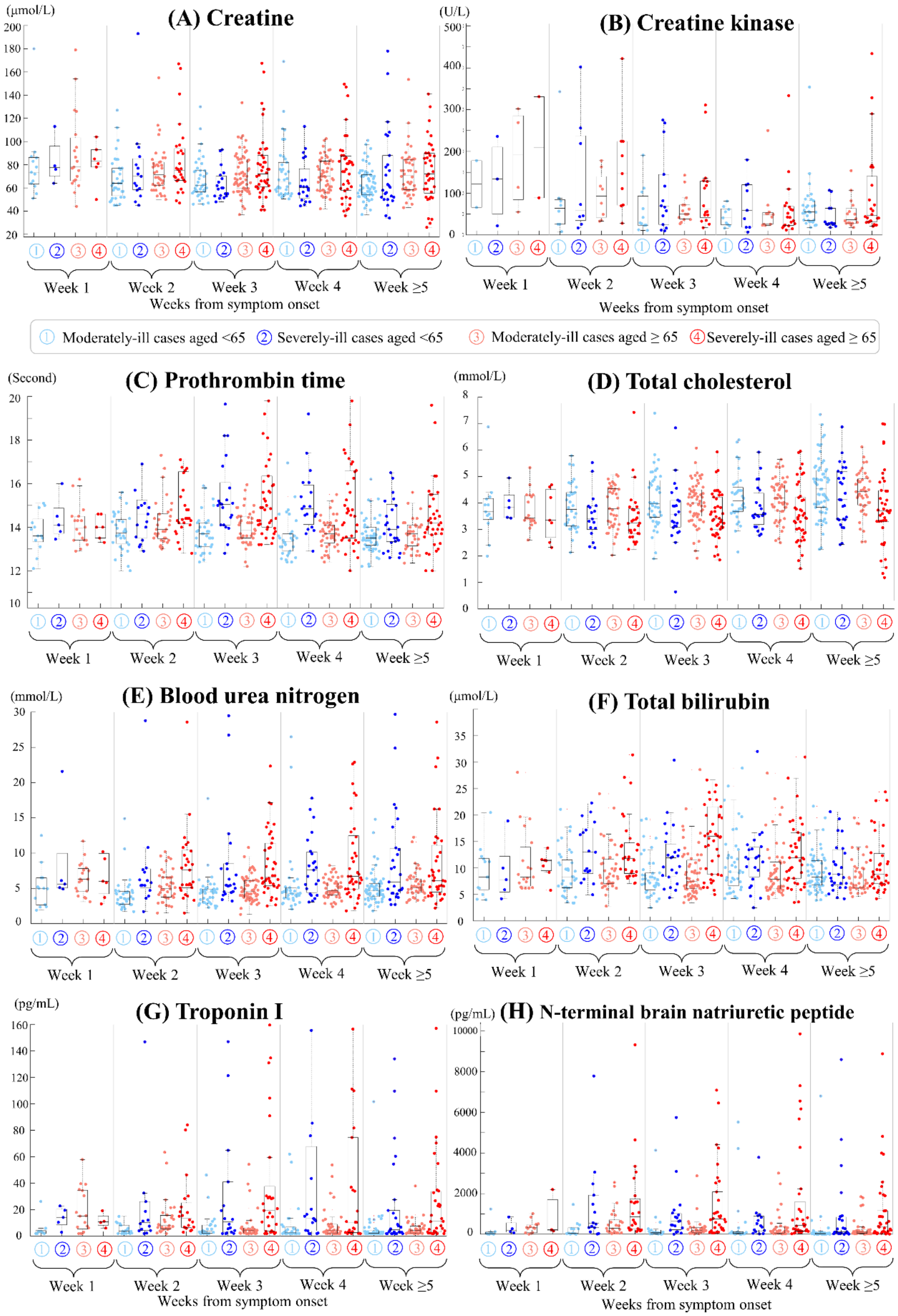

Figure S2: Dynamics of creatine (A), creatine kinase (B), prothrombin time (C), total cholesterol (D), urea nitrogen (E), total bilirubin (F), troponin I (G), N-terminal brain natriuretic peptide **(H) in COVID-19 patients.** Four groups were visualized in light blue (moderately-ill patients aged <65 years), blue (severely-ill patients aged <65 years), light red (moderately-ill patients aged ≥65 years), and red (severely-ill patients aged ≥65 years).

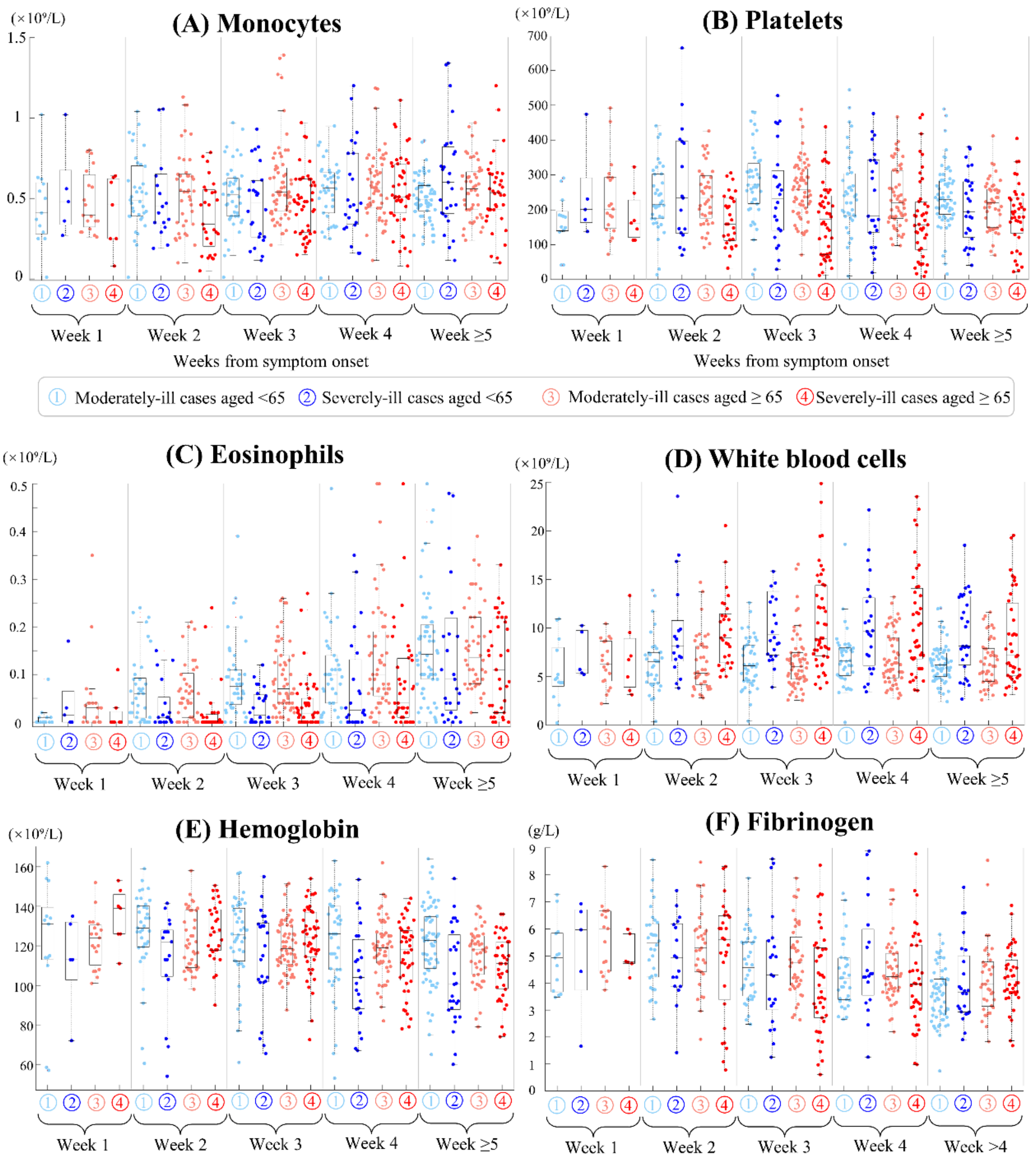

Figure S3: Dynamics of monocytes (A), platelets (B), eosinophils (C), white blood cells (D), hemoglobin (E), and fibrinogen (F) in COVID-19 patients. Four patient groups were visualized in light blue (moderately-ill patients aged <65 years), blue (severely-ill patients aged <65 years), light red (moderately-ill patients aged ≥65 years), and red (severely-ill patients aged ≥65 years).

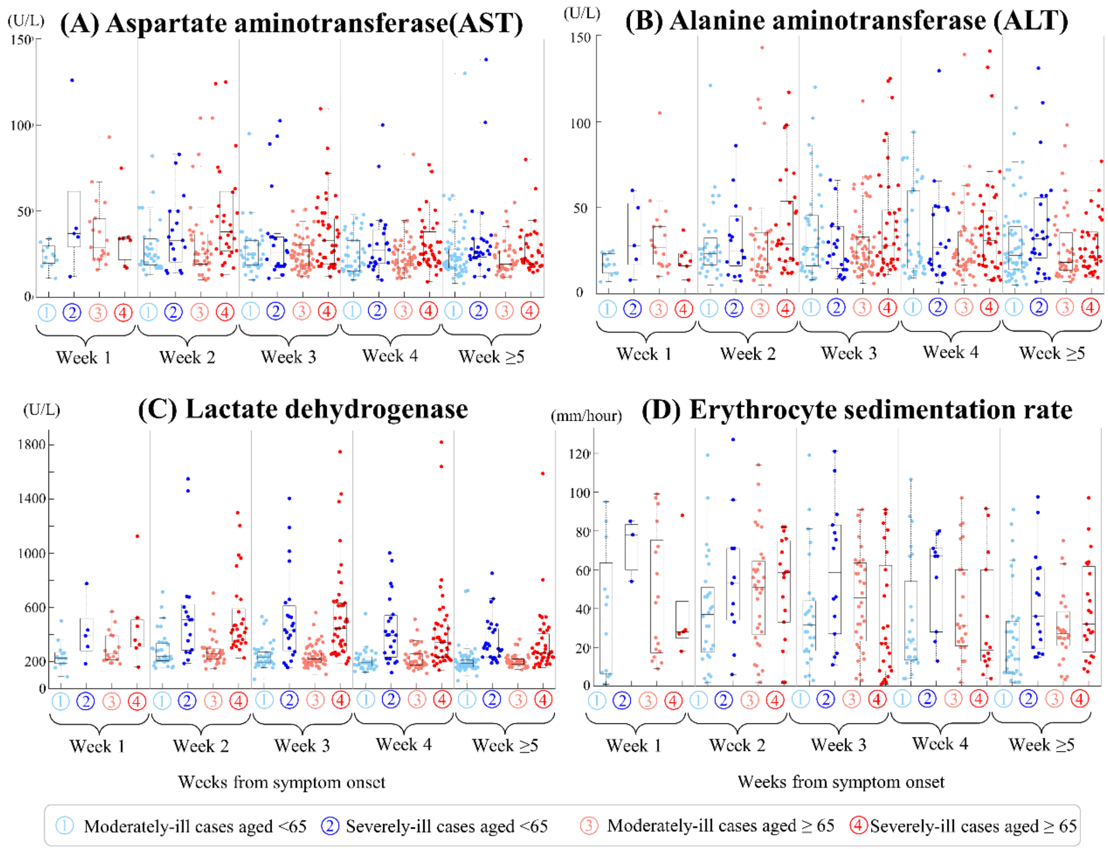

Figure S4: Dynamics of aspartate aminotransferase (A), alanine aminotransferase (B), lactate dehydrogenase (C), and erythrocyte sedimentation rate (D) in COVID-19 patients. Four patient groups were visualized in light blue (moderately-ill patients aged <65 years), blue (severely-ill patients aged <65 years), light red (moderately-ill patients aged ≥65 years), and red (severely-ill patients aged ≥65 years).

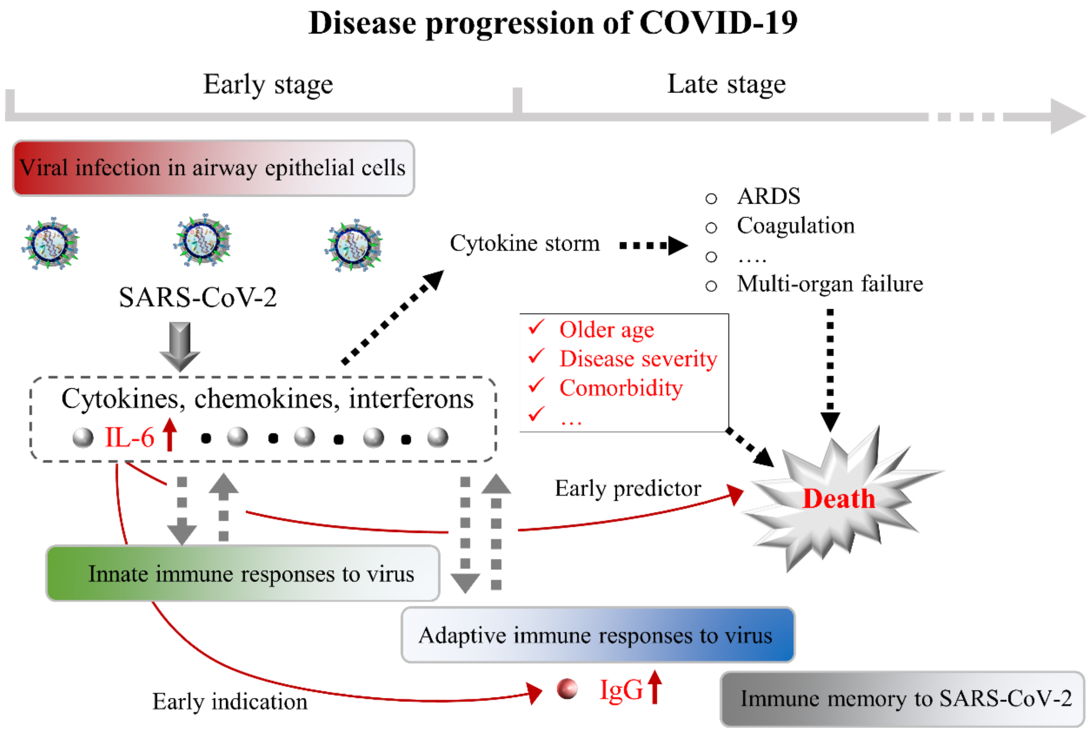

Figure S5: Our hypothesis of the cytokine - antibody associations during the disease progression of COVID-19. This figure is adapted from previous publications [1-4]. Briefly, the disease progression of SARS-CoV-2 can be intuitively divided into the early stage (nearly 2 weeks after symptom onset) and the late stage (>2 weeks after symptom onset) [1]. During the early stage, SARS-CoV-2 infects airway epithelial cells with the surface receptors such as ACE2 and TMPRSS2 [3]. The active replication and release of viral particles cause host cells to trigger the generation of pro-inflammatory cytokines (e.g., IL-6, IL-10, TNF), chemokines (e.g., CCL2, CCL3, CCL5, CXCL10), and interferons (type I/III interferons) [1, 3]. This attracts monocytes, macrophages, and T cells to the infected cells and establish a pro-inflammatory feedback loop. The innate immunity responses to SARS-CoV-2 by activating many signaling pathways during the early stage of infection, while the adaptive immune responses take over during the late stage with production of antibodies such as anti-SARS-CoV-2 IgG [2]. The defective immune response causes the overproduction of cytokines, resulting in cytokine storm that causes fatal symptoms such as acute respiratory distress syndrome (ARDS), severe pneumonia, multiorgan failure, and coagulation damage [3, 4]. The red lines indicate findings of our study that early responses of cytokines are associated with IgG responses at the late stage, and baseline cytokines and other factors such as older age can be early predictors of death outcome.

References

1. Schultze JL, Aschenbrenner AC: **COVID-19 and the human innate immune system.** *Cell* 2021, **184:**1671-1692.
2. Cox RJ, Brokstad KA: **Not just antibodies: B cells and T cells mediate immunity to COVID-19.** *Nat Rev Immunol* 2020, **20:**581-582.
3. Tay MZ, Poh CM, Renia L, MacAry PA, Ng LFP: **The trinity of COVID-19: immunity, inflammation and intervention.** *Nat Rev Immunol* 2020, **20:**363-374.
4. Hojyo S, Uchida M, Tanaka K, Hasebe R, Tanaka Y, Murakami M, Hirano T: **How COVID-19 induces cytokine storm with high mortality.** *Inflamm Regen* 2020, **40:**37.
